# Supplementary material for: Fractional Chern insulators in magic-angle twisted bilayer graphene
Source: Nature. 2021 Dec 15;600(7889):439–43. doi: 10.1038/s41586-021-04002-3 (PMC8674130; doi:10.1038/s41586-021-04002-3)
Supplement: Supplementary file 1 — This file has two sections. The first describes the relation between the stability of FCIs and the quantum geometry of twisted BLG. The second section is entirely technical and gives the complete mathematical and numerical details of our models. [file 41586_2021_4002_MOESM1_ESM.pdf]

nature portfolio

https://doi.org/10.1038/s41586-021-04002-3

Supplementary information

Fractional Chern insulators in magic-angle twisted bilayer graphene

In the format provided by the authors and unedited

Supplemental Information: Fractional Chern insulators in magic-angle twisted bilayer graphene

This Supplemental Information has two sections. The first describes the relation between the stability of fractional Chern insulators and the quantum geometry of twisted bilayer graphene. The second section is entirely technical and gives the complete mathematical and numerical details of our models.

I. BAND GEOMETRY OF FRACTIONAL CHERN INSULATORS

Just as Chern insulators generalize the integer quantum hall effect from the lowest Landau level (LLL) to a non-trivial band stucture, fractional Chern insulators (FCIs) upgrade the fractional quantum hall effect (FQHE) to the lattice setting. FCIs have only discrete translation symmetry and, in principle, do not require an external magnetic field, but enjoy the other topological properties of the FQHE. In hopes of finding such an intriguing phase, a large number of theoretical and numerical works have considered what conditions a model must satisfy to support an FCI phase (see e.g. [1–10]). One main finding is that, as most FCI states are adiabatically connected to the FQHE [3, 11], they generally satisfy three conditions:

C1. Topologically, the parent state must be a Chern insulator with $|C| > 0$.

C2. Energetically, the "host" band(s) for the FCI should be strongly interacting, with

$$W \ll V \ll \Delta E, \tag{S1}$$

where W is their bandwidth, V is the interaction strength, and ΔE is the gap to any other bands.

C3. Geometrically, the quantum geometry should be "close enough" to the lowest Landau level. The distance from the ideal geometry is often quantified by the following figures of merit

$$\begin{aligned} 0 &\leq \sigma[\mathcal{F}], && \text{(Berry curvature deviation)} \\ 0 &\leq T[\eta], && \text{(Trace deviation)} \end{aligned}$$

which we define carefully below.

All three conditions are properties of the band structure alone, yet they constitute strong predictors for when the many-body ground state is an FCI. We may therefore make predictions for where FCIs are likely to appear without solving the full many-body problem. In the remainder of this section we define the band geometry, then examine the case of magic-angle twisted bilayer graphene (TBG). We find that hBN-aligned TBG provides a good parent state satisfying C1 and C2, but that the conditions in C3 are slightly too large at zero field. Finally, we show that adding a field reduces $\sigma[\mathcal{F}]$, $T[\eta]$, and the bandwidth, likely stabilizing an FCI phase.

A. Quantum Band Geometry Definitions

We now define the quantum band geometry. As there are many minibands at finite field, we present a natural many band generalization of condition C3 which is continuous, gauge-invariant, and reduces to the expected value at $\Phi/\Phi_0 \to 0$.

Consider a single particle bandstructure with Bloch wavefunctions $h(\boldsymbol{k})\,|u_{\boldsymbol{k}a}\rangle = \epsilon_{\boldsymbol{k}a}\,|u_{\boldsymbol{k}a}\rangle$. Suppose there are $N \geq 1$ "host" bands which are well-separated from the rest by a gap.[1] Define the projector

$$\mathcal{P}_{\boldsymbol{k}} \;:=\; \sum_{a=1}^{N} |u_{\boldsymbol{k}a}\rangle\,\langle u_{\boldsymbol{k}a}|\,, \tag{S3}$$

which is gauge-invariant under all $U(N)$ transformations of the host bands. The quantum geometry is entirely determined by $\mathcal{P}_{\boldsymbol{k}}$ [12]. Define the non-Abelian $U(N)$ quantum geometric tensor (or "QGT", see e.g. [2, 12, 13])

$$\eta^{\mu\nu}_{ab}(\boldsymbol{k}) := NA\,\langle \partial^\mu u_{\boldsymbol{k}a}|(1-\mathcal{P}_{\boldsymbol{k}})|\partial^\nu u_{\boldsymbol{k}b}\rangle \tag{S4}$$

---

[1] Generically, N is a function of field.

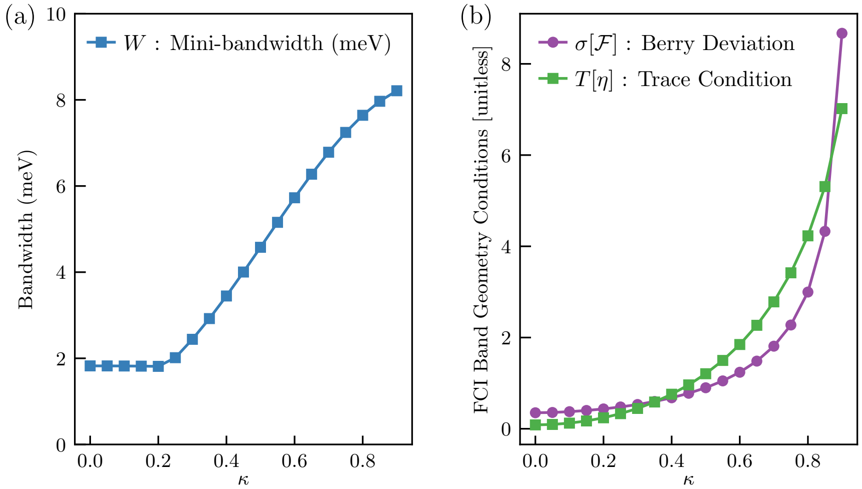

FIG. S1. (a) Bandwidth and (b) Band geometry indicators of FCI stability in the Bistritzer-MacDonald model as a function of κ with an hBN mass of 30 meV, $\theta = 1.06^\circ$, $w_1 = 110$ meV. The bandwidth does not vanish at $\kappa = 0$ since θ is slightly detuned from the magic angle.

where $\partial^\mu = \frac{\partial}{\partial k^\mu}$, and $A = A(\Phi/\Phi_0)$ is the area of the (magnetic) Brillouin zone. (The non-standard factor of NA makes η dimensionless.) This is a Hermitian metric whose symmetric and antisymmetric parts

$$\mathcal{F}(\boldsymbol{k}) = -\varepsilon_{\mu\nu}\eta^{\mu\nu}(\boldsymbol{k}), \tag{S5}$$

$$g^{\mu\nu}(\boldsymbol{k}) = \frac{1}{2}\left(\eta^{\mu\nu}(\boldsymbol{k}) + \eta^{\nu\mu}(\boldsymbol{k})\right) \tag{S6}$$

are the quantum (Fubini-Study) metric and Berry curvature respectively.

We emphasize that g plays an important role in interacting physics of flat bands. When $N = 1$, the quantum metric is a 2×2 real, symmetric matrix, which encodes the distance between Bloch states:

$$|\langle u_{\boldsymbol{k}}|u_{\boldsymbol{k}+d\boldsymbol{k}}\rangle| = 1 - \sum_{\mu,\nu=1}^{2} g^{\mu\nu}(\boldsymbol{k})\, dk_\mu dk_\nu. \tag{S7}$$

All Landau levels have the same, flat, Berry curvature and the same bandwidth of zero, but are distinguished by their metrics[2]

$$g^{(n)}_{\mu\nu}(\boldsymbol{k}) = \left(n + \frac{1}{2}\right)\delta_{\mu\nu}. \tag{S8}$$

It is crucial to note that the QGT is independent of the energetics; one can alter $h(\boldsymbol{k})$ without changing $\eta^{\mu\nu}_{ab}(\boldsymbol{k})$. A uniform $\eta(\boldsymbol{k})$ across the Brillouin zone does *not* imply that the associated band is flat. Furthermore, in the non-Abelian case, g and $\mathcal{F}$ are only gauge-covariant, not gauge-invariant. The individual components of $\mathcal{F}_{ab}$ are gauge-dependent, so only the trace, determinant, and eigenvalues of $\mathcal{F}$ are observable.

To evaluate expectations of the Berry curvature distribution, define the scaled trace

$$\mathrm{Tr}[\mathcal{O}] := (NA)^{-1}\sum_{b=1}^{N}\int d^2\boldsymbol{k}\ \mathcal{O}_{bb}(\boldsymbol{k}) \tag{S9}$$

so that $\mathrm{Tr}[\mathrm{Id}] = 1$. The normalization is selected so all moments of the Berry curvature distribution are dimensionless, i.e. so the measure is dimensionless. For instance, the Chern number

$$C := \frac{1}{2\pi}\,\mathrm{Tr}[\mathcal{F}] \tag{S10}$$

---

[2] The term proportional to n comes from the Laguerre polynomial in the n'th Landau level form factor $|\langle u_{\boldsymbol{k}}|u_{\boldsymbol{k}+\boldsymbol{q}}\rangle| = L_n(q^2\ell_B^2/2)\exp(-q^2\ell_B^2/4)$. The form factor has a node due to the Laguerre polynomial which favors charge density wave states in higher Landau levels [14].

is simply the mean of the Berry curvature distribution, divided by 2π.

In the lowest Landau level, the quantum geometric tensor is (see e.g. [15]):

$$\eta(\boldsymbol{k}) = \frac{1}{2}\begin{pmatrix} 1 & i \\ -i & 1 \end{pmatrix}, \tag{S11}$$

for $B > 0$ and $A = \ell_B^2 = \hbar/(eB)$. In many ways, Eq. (S11) is the simplest topologically nontrivial band geometry, and obeys a number of special conditions:

- The Berry curvature $\mathcal{F}(\boldsymbol{k}) = 1$ is completely uniform with Chern number $C = A^{-1}\int_A d^2\boldsymbol{k}\,\mathcal{F}(\boldsymbol{k}) = 1$.
- The quantum geometric tensor saturates the inequality

$$\det \eta \geq 0, \tag{S12}$$

  known as the *determinant condition* or *ideal droplet condition.*
- Even though (S12) is already a strong constraint, it obeys the strictly stronger *isotropic ideal droplet condition* or *trace condition.* Namely, it saturates the inequality

$$\operatorname{tr} g(\boldsymbol{k}) - |\mathcal{F}(\boldsymbol{k})| \geq 0. \tag{S13}$$

Note that higher Landau levels satisfy neither the determinant condition nor the trace condition. There is a close and intriguing connection between the trace condition, the GMP algebra, and Kahler geometry, which makes clear that these properties are not accidents, but instead essential structural facets of lowest Landau levels [2, 13, 15, 16].

To assess how far the quantum band geometry of a generic band structure is from the LLL limit, we measure the failure of these conditions. In particular, we quantify the non-uniformity of the Berry curvature via the standard deviation of the Berry curvature distribution:

$$\sigma[\mathcal{F}] := \operatorname{Tr}\left[\left(\mathcal{F}/(2\pi) - C\right)^2\right]^{1/2}. \tag{S14}$$

Similarly, we quantify the failure of the trace condition by

$$T[\eta] := \operatorname{Tr}\left[\operatorname{tr} g - |\mathcal{F}|\right]. \tag{S15}$$

One can show that $T[\eta]$ vanishes if, and only if, the Bloch wavefunctions are meromorphic functions of $k_x + ik_y$. In fact, if both $\sigma[\mathcal{F}] = 0$ and $T[\eta] = 0$, then the band geometry is identical to that of the Lowest Landau level.

B. Band Geometry of TBG at Zero Field

At zero field, the BM model [17] with hBN-alignment satisfies conditions C1 and C2 and, at experimental parameters, nearly satisfies C3. Alignment to hBN gives a sublattice potential, giving the narrow bands Chern number $C = \pm 1$. Their bandwidth is much less than the interaction strength, and they are well-separated from the remote bands (see Fig. S2). In other words, conditions C1 and C2 hold. Therefore TBG should provide a topologically and energetically suitable parent state for a FCI.

Due to this promising starting point, a number of studies have examined the problem both analytically [7] and numerically [8–10]. They found that a primary parameter which controls the physics is $\kappa = w_0/w_1$, the ratio of interlayer tunneling on AA versus AB/BA sublattices. In the so-called chiral limit $\kappa = 0$, the non-interacting ground state is exactly a LLL in a spatially-inhomogenous magnetic field [7], so C1-C3 hold exactly except for a small Berry curvature inhomogeneity. A Laughlin state may be constructed explicitly, and a pseudopotential argument then shows that the ground state of the interacting Hamiltonian is an FCI. As κ increases, $\sigma[\mathcal{F}]$ and $T[\eta]$ increase slowly at first, but then quickly beyond $\kappa \approx 0.6$ (see Fig. S1). The numerical studies, summarized in Table I, indicate that we expect a transition out of an FCI state around $\kappa \approx 0.7$, which is within the range predicted by density functional theories [18]. No FCI is observed in the experiment at zero field, suggesting that the sample is barely beyond the transition.

We note that the bandwidth, as well as the band geometry, can also limit FCI formation through condition C2. Fig. S1 shows the bandwidth W as a function of κ. Since the interaction strength V is expected to be on the ~ 20 meV level, W/V is a good perturbative parameter at the single-particle level. An important caveat is that the bandwidth is expected to be renormalized substantially by interactions, possibly violating condition C2. Considering only the 'bare' single-particle bandwidth may therefore provide a bias towards FCI formation. However, the band geometry indicators increase sharply with κ, while the bandwidth increases only gradually. The numerical results therefore suggest that band geometry is a driving factor as to whether an FCI is realized, particularly $\sigma[F]$, which varies strongly with κ. The band geometric quantities $\sigma[\mathcal{F}]$ and $T[\eta]$ appear to be only slightly too large at realistic κ — and if they could be reduced then an FCI would likely be stabilized.

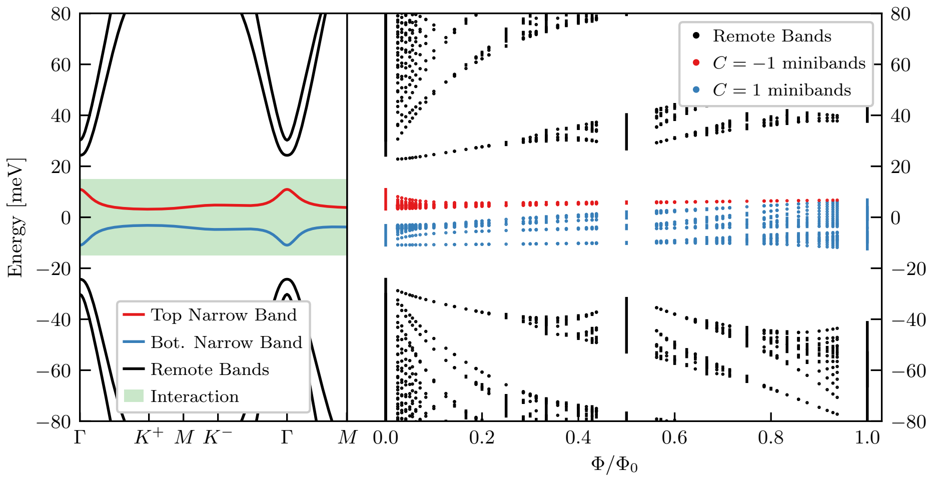

FIG. S2. (Left) Spectrum of the Bistritzer-MacDonald model with hBN alignment. The green band shows the approximate strength of Coulomb interactions. Parameters: $\kappa = 0.8$, $\theta = 1.06°$, $\Delta_{\mathrm{hBN}} = 30\,\mathrm{meV}$ for the bottom layer only. (Right) Spectrum of the same model in a finite perpendicular magnetic field, computed at fractions $\Phi/\Phi_0 = p/(2q)$ for $q \leq 8$. Numerical details are given in Sec. II.

Reference	hBN?	Valley Pol.	Spin Pol.	Kinetic Energy	w_1 [meV]	Chern #	κ_c
Repellin & Senthil [8]	Yes	Yes	No	$<$ (1/10) BM	110	1	0.9
Abouelkomsan *et al.* [9]	Yes	Yes	Yes	HF-corrected	90	1	> 0.7
	Yes	Yes	Yes	HF-corrected	110	1	> 0.7
Wilhelm *et al.* [10]	Yes	Yes	Yes	BM	90	1	> 0.7
	Yes	Yes	Yes	BM	110	1	< 0.7

TABLE I. Selected numerical studies of fractional Chern insulators in bilayer graphene. Assuming that FCIs are always stabilized at low κ, the last column indicates where the transition κ_c might lie. Here HF-corrected means that the kinetic energy is modified to account for some Hartree-Fock corrections.

C. Band Geometry of TBG at Finite Field

At a finite magnetic field, the spectrum of the BM model develops a fractal structure (see Fig. S2) known as the Hofstadter butterfly [19]. Significantly, the magnetic Brillouin zone is only well-defined when the flux through the unit cell is a rational number

$$\phi := \frac{\Phi}{\Phi_0} = \frac{BA}{h/e} \equiv \frac{p}{q} \in \mathbb{Q}. \tag{S16}$$

The resulting magnetic unit cell is q times larger than the original in real space, and thus q times smaller in reciprocal space — but with q times as many bands as the original model. The fact that changing the flux changes the Brillouin zone leads to a number of difficulties, and some quantities are no longer well-defined. The remedy is to consider sets of *minibands*, groups of bands with a gap both above and below for a finite range of flux. Intrinsic properties of the minibands, such as their total Chern number, are well-defined and a continuous function of field.

The $C = -1$ host minibands (red in Fig. S2) are separated from the rest of the spectrum by a gap for $0 \leq \phi < 1$; one can define functions $E_b(\phi) < \epsilon < E_t(\phi)$ which bound all eigenvalues ϵ in the top minibands. Explicitly, one can define a projector

$$\mathcal{P}(\phi) := \sum_{E_b(\phi)<\epsilon<E_t(\phi)} |\epsilon\rangle\langle\epsilon| = \sum_{a=1}^{N(\phi)} \sum_{\boldsymbol{k}} |u_{\boldsymbol{k}a}\rangle\langle u_{\boldsymbol{k}a}| \tag{S17}$$

to the top set of N_ϕ host minibands. Using this projector together with the normalization conventions in Eq. (S9) ensures that the $\sigma[\mathcal{F}]$ and $T[\eta]$ are gauge-invariant, continuous functions of flux, and reduce to the expected value as $\phi \to 0$. They are shown in Fig. S3, along with the single-particle bandwidth. One striking observation is that $\sigma[\mathcal{F}] \to 0$ and $T[\eta] \to 0$ as $\phi \to 1$, so the single particle wavefunctions become those of the lowest Landau level.

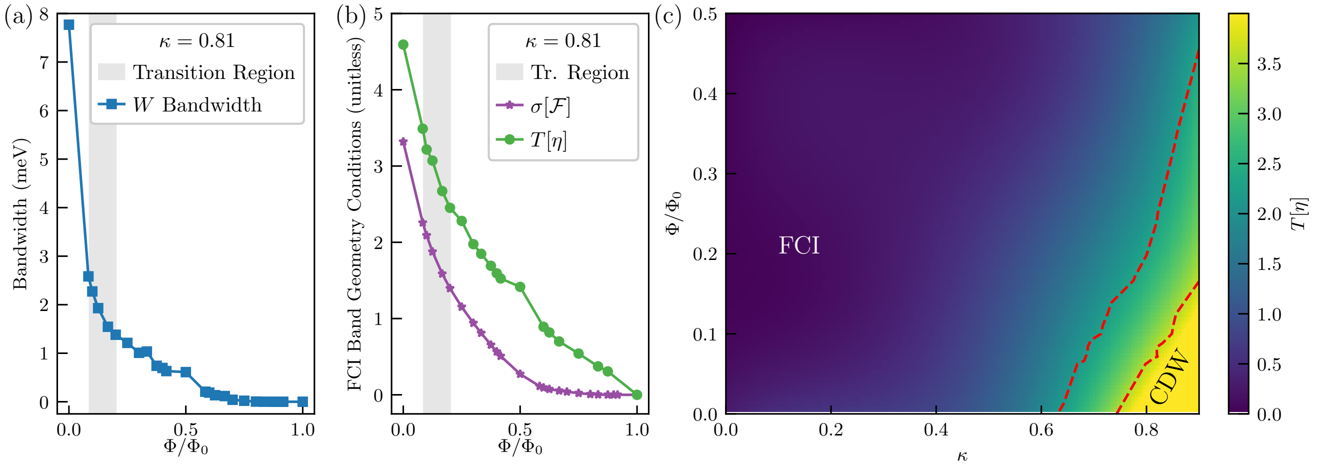

FIG. S3. (a) Bandwidth of the set of "host" minibands (highlighted red in Fig. S2) as a function of flux. The gray band indicates the lowest flux at which an FCI is observed experimentally. (b) Band geometry indicators, (Berry curvature standard deviation $\sigma[\mathcal{F}]$ and trace condition violation $T[\eta]$) as a function of flux. (c). The trace condition $T[\eta]$ as a function of both κ and field. The dashed red lines are are values that intersect $\phi = 0$ at $\kappa = 0.65, 0.75$, demarcating the approximate transition region. The labels FCI and CDW (charge density wave) mean the quantum geometry is favorable for those phases to appear. Numerical details are given in Sec. II. Parameters: $\theta = 1.06^\circ$, $\Delta_{\mathrm{hBN}} = 30\,\mathrm{meV}$.

We underscore that, in the region where FCIs are observed experimentally ($\phi \gtrsim 0.2$), the band geometry is quite dissimilar to the LLL. Physically, the FCIs are far from the $\phi \approx 1$ LLL, and are mostly governed by the parent Chern bands at $\phi = 0$.

We may use the band geometry conditions to roughly sketch the many-body phase diagram. To "calibrate" the meaning of the band geometry conditions, we consider the exact many-body $B = 0$ results. Table I provides estimates of the values of $\sigma[\mathcal{F}]$ and $T[\eta]$ at which the FCI becomes destabilized. If we assume that these values also delineate the region of stability at finite field, then choosing a range $\sigma[\mathcal{F}] = 1.4 - 2.2$—consistent with the experiment and with the theoretical estimates—produces the phase diagram in Fig. 3g of the main text. These values should not be interpreted as more than rough indicators of where a many-body phase transition might occur. For instance, the FCI could in principle be destabilized by bandwidth rather than quantum geometry. Furthermore, the exact diagonalization results choose parameters such that the bandwidth is reduced from its physical value, biasing the results in favor of forming an FCI. Nevertheless, we conclude that the experimental results are consistent with the interpretation that the perpendicular field reduces the "effective κ" of TBG, favoring fractional Chern insulators at relatively small field.

II. CONSTRUCTION OF THE TBG HAMILTONIAN IN A FINITE MAGNETIC FIELD

In this section we construct a model for twisted bilayer graphene in a finite magnetic field. The strategy is to project the standard Bistrizter-MacDonald model into a basis of Landau levels (instead of plane waves). Similar models have been considered in [20–22], but here we take a more pedagogical approach.

We take the unit cell convention

$$\boldsymbol{q}_1 = k_\theta(0,-1), \quad \boldsymbol{q}_{2,3} = k_\theta(\pm\sqrt{3}/2, 1/2), \qquad k_\theta = \frac{8\pi}{3\sqrt{3}a}\sin(\theta/2), \tag{S18}$$

where a is the carbon-carbon bond length in graphene. The moiré unit cell and Brillouin zone are spanned by

$$\boldsymbol{a}_{1,2} = \frac{4\pi}{3k_\theta}(\pm\sqrt{3}/2, 1/2); \quad \boldsymbol{b}_{1,2} = k_\theta(\pm\sqrt{3}/2, 3/2) \tag{S19}$$

such that $\boldsymbol{a}_i \cdot \boldsymbol{b}_j = 2\pi\delta_{ij}$.

The standard BM model [17] is defined in real space as a 4×4 matrix in layer-sublattice space, which we index with Pauli matrices γ and σ, respectively. It consists of a kinetic part for the Dirac cones of the two layers, and an

interlayer tunneling term:

$$H(\boldsymbol{r}) = \begin{pmatrix} h^+ & T(\boldsymbol{r}) \\ T(\boldsymbol{r})^\dagger & h^- \end{pmatrix}, \quad h^\pm = -i\hbar v_F \boldsymbol{\sigma}_{\pm\theta/2} \cdot \left[\boldsymbol{\nabla} \mp \frac{i}{2}\boldsymbol{q}_1\right], \quad T(\boldsymbol{r}) = \sum_{s=0}^{2} T_s e^{-i\boldsymbol{b}_s\cdot\boldsymbol{r}}, \quad T_s = \begin{pmatrix} w_0 & \zeta^s w_1 \\ \zeta^{-s} w_1 & w_0 \end{pmatrix}, \tag{S20}$$

where $\boldsymbol{\sigma}_{\theta/2} = e^{-i\theta\sigma_z/4}\boldsymbol{\sigma}e^{i\theta\sigma_z/4}$, $\zeta = e^{i2\pi/3}$, $\boldsymbol{b}_0 = 0$, $\boldsymbol{q}_1 = k_\theta(0,-1)$ is the offset of the Dirac point, and v_F is the Fermi velocity of graphene.

To introduce a magnetic field, we employ the Pierls substitution $\boldsymbol{p} \to \boldsymbol{\pi} = \boldsymbol{p} + e\boldsymbol{A}$. Schematically the "Magnetic BM model" is still a sum of a kinetic term and a periodic potential: $H_{MBM} = H_K + H_V$. To employ the model for useful computations, however, one must put it in the right basis — a task complicated by the magnetic field. An immediate difference is that the eigenstates of the kinetic term are now Landau levels instead of plane waves. Furthermore, the magnetic field dramatically alters the structure of translation-invariance. Only at rational values of flux through a unit cell do we recover a mutually commuting pair of magnetic translation vectors and, hence, the magnetic Brillouin zone. Below we review the physics of Landau levels and derive a few useful identities they satisfy, then describe the magnetic translation algebra and how to define the Bloch wavefunctions in the presence of magnetic field. After that, constructing the "magnetic BM model" is a straightforward (albeit involved) change of basis.

A. Landau Levels

We start off by recalling the physics of Landau levels, using conventions from [23]. The free electron has canonical commutation relations $[r_\alpha, r_\beta] = 0 = [p_\alpha, p_\beta]$, $[r_\alpha, p_\beta] = i\hbar\delta_{\alpha\beta}$, with $p_\alpha = -i\hbar\partial_\alpha$. If we introduce a magnetic field by $\boldsymbol{p} \to \boldsymbol{\pi} = \boldsymbol{p} + e\boldsymbol{A}$, then components of momenta no longer mutually commute:

$$[\pi_x, \pi_y] = -ie\hbar B = -i\frac{\hbar^2}{\ell^2}, \tag{S21}$$

where we have introduced the magnetic length $\ell = \sqrt{\frac{\hbar}{eB}}$. However, momenta do commmute with the guiding center coordinates

$$\begin{cases} R_x & := x - \frac{\ell^2}{\hbar}\pi_y \\ R_y & := y + \frac{\ell^2}{\hbar}\pi_x \end{cases} \tag{S22}$$

so that $[R_\alpha, \pi_\beta] = 0$. The guiding centers also fail to commute among themselves: $[R_x, R_y] = i\ell^2$.

In the presence of a magnetic field, the free electron eigenstates are no longer planewaves, but instead Landau levels. Define raising and lowering operators (Landau levels) as

$$\hat{a}^\pm := \frac{\ell}{\sqrt{2}\hbar}\left(\pi_x \pm i\pi_y\right), \tag{S23}$$

which satisfy $[\hat{a}, \hat{a}^\dagger] = 1$. The free electron becomes $H_0 = \pi^2/(2m) = \hat{a}^\dagger\hat{a} + \frac{1}{2}$ with $m = 1/(eB\hbar)$, which is a quantum harmonic oscillator. Furthermore, as $[\hat{a}^\pm, R_\alpha] = 0$, we may choose either one of the two guiding center coordinates as a good quantum number.

To write explicit wavefunctions, we choose the Landau gauge $A = (-By, 0)$, so that $\pi_x = p_x - eBy$ and $\pi_y = p_y$. We select $Y := R_y = \frac{\ell^2}{\hbar}p_x =: \ell^2 k_Y$ as a good quantum number, and the lowering operator becomes

$$\frac{\sqrt{2}}{\hbar\ell}\hat{a} = \frac{1}{\hbar}(\pi_x - i\pi_y) = \frac{1}{\hbar}(p_x + eA_x - ip_y) = k_Y - \frac{eB}{\hbar}y - \partial_y = -\partial_y - [y - \ell^2 k_Y]/\ell^2. = -\partial_y - [y - Y]/\ell^2 \tag{S24}$$

It is often convenient to work in dimensionless units for harmonic oscillator computations. When working at a fixed k_Y, we define $\tilde{y} = [y - Y]/\ell$ and $\tilde{p}_y = -i\partial_{\tilde{y}}$, so that $\hat{a} = -\frac{1}{\sqrt{2}}[\tilde{y} + i\tilde{p}_y]$.

The wavefunctions are therefore planewaves in the x-direction and harmonic oscillator wavefunctions in the y-direction:

$$\langle x, y | n, Y = \ell^2 k_Y\rangle = e^{ik_Y x}\varphi_{nk_Y}(y); \quad \varphi_{nk_Y}(y) := \ell^{-1/2}\varphi_n([y - Y]/\ell), \tag{S25}$$

$$\langle y | n\rangle = \varphi_n(y) := \left(2^n n! \sqrt{\pi}\right)^{-1/2} H_n(y) e^{-y^2/2} \tag{S26}$$

where H_n are the Hermite polynomials as usual. The operators $\hat{a}^{\pm}$ raise and lower the Landau level index n. Below we will use these as the basis states for TBG at finite field.

We now derive an identity we will use several times below. We wish to evaluate

$$\mathcal{M}_{mn}(\boldsymbol{q}) = \int_{\mathbb{R}} d\tilde{y}\, \varphi_m^*(\tilde{y}) e^{-iq_y\ell\tilde{y}} e^{-iq_x\ell\tilde{p}_y} \varphi_n(\tilde{y}) = \langle m| e^{-iq_y\ell\tilde{y}} e^{-iq_x\ell\tilde{p}_y} |n\rangle\,. \tag{S27}$$

By Baker-Campbell-Hausdorff,

$$e^{-iq_y\ell\tilde{y}} e^{-iq_x\ell\tilde{p}_y} = e^{-iq_xq_y\ell^2/2} e^{-iq_x\ell\tilde{p}_y - iq_y\ell\tilde{y}} = e^{-iq_xq_y\ell^2/2} e^{z^*\hat{a}^\dagger - z\hat{a}}, \quad z := \frac{\ell}{\sqrt{2}}(q_x + iq_y). \tag{S28}$$

So $\mathcal{M}_{mn}(\boldsymbol{q}) = e^{-iq_xq_y\ell^2/2} \langle m| e^{z^*\hat{a}^\dagger - z\hat{a}} |n\rangle$. The remaining matrix element is essentially a displacement operator, whose matrix elements we now evaluate. Another application of Baker-Campbell-Hausdorff gives

$$e^{z^*\hat{a}^\dagger - z\hat{a}} = e^{z^*\hat{a}^\dagger} e^{-z\hat{a}} e^{-|z|^2/2}.$$

Since $e^{z^*\hat{a}^\dagger} e^{-za}$ is normal ordered, it becomes a finite sum over intermediate states $|\ell\rangle$ with $\ell < n, m$, which may be expressed in terms of the Laguerre polynomials.

$$\langle m| e^{z^*\hat{a}^\dagger} e^{-z\hat{a}} |n\rangle = \sum_{\ell,N,M=0}^{\infty} \left\langle m \middle| \frac{(z^*)^M}{M!} (\hat{a}^\dagger)^M \middle| \ell \right\rangle \left\langle \ell \middle| \frac{(-z)^N}{N!} \hat{a}^N \middle| n \right\rangle \tag{S29}$$

$$= \sum_{\ell,M,N} \frac{(z^*)^M}{M!} \frac{(-z)^N}{N!} \delta_{m,M+\ell} \sqrt{\frac{m!}{\ell!}} \delta_{\ell+N,n} \sqrt{\frac{n!}{\ell!}} \tag{S30}$$

$$= \sum_{0\le\ell\le n,m} \frac{(z^*)^{m-\ell}}{(m-\ell)!} \frac{(-z)^{n-\ell}}{(n-\ell)!} \sqrt{\frac{m!\,n!}{\ell!\,\ell!}} \tag{S31}$$

where the last step used the δ's. Now suppose that $m \ge n$ and let $k = n - \ell$. Then this becomes

$$\sum_{k=0}^{n} \frac{(z^*)^{m-n+k}}{(m-n+k)!} \frac{(-z)^k}{k!} \frac{\sqrt{m!\,n!}}{(n-k)!} \sqrt{\frac{m!}{m!}} = \sqrt{\frac{n!}{m!}} (z^*)^{m-n} \sum_{k=0}^{n} (-1)^k \frac{(m-n+n)!}{(m-n+k)!\,k!\,(n-k)!} (zz^*)^k \tag{S32}$$

In terms of the associated Laguerre polynomials

$$L_n^\alpha(x) = \sum_{k=0}^{n} (-1)^k \frac{(n+\alpha)!}{(k+\alpha)!\,k!\,(n-k)!} x^k, \tag{S33}$$

we have

$$L_{mn}(z) := \langle m| e^{z^*\hat{a}^\dagger - z\hat{a}} |n\rangle = \begin{cases} \sqrt{\frac{n!}{m!}} (z^*)^{m-n} L_n^{m-n}(|z|^2) e^{-|z|^2/2} & \text{if } m \ge n \\ \sqrt{\frac{m!}{n!}} (-z)^{n-m} L_m^{n-m}(|z|^2) e^{-|z|^2/2} & \text{if } m < n. \end{cases} \tag{S34}$$

The L's obey $L_{mn}(z^*) = L_{mn}(z)^*$, $L_{mn}(-z) = L_{nm}(z)^*$ and $L_{mn}(0) = \delta_{mn}$. Combining everything,

$$\mathcal{M}_{mn}(\boldsymbol{q}) = \langle m| e^{-iq_y\ell\tilde{y}} e^{-iq_x\ell\tilde{p}_y} |n\rangle = e^{-iq_xq_y\ell^2/2} L_{mn}(z) \qquad z := \frac{\ell}{\sqrt{2}}(q_x + iq_y). \tag{S35}$$

This identity will prove useful below.

B. Magnetic Brillouin Zone and Magnetic Basis

Next we review how Bloch's theorem must be generalized in the presence of a magnetic field, and how to choose the right basis for our model. It was recognized long ago by Zak [24, 25] that the group of (lattice) translations is modified in the presence of a magnetic field and becomes non-commutative. We shall see that only rational amounts

of flux lead to mutually commuting translation operators and thence to a magnetic version of Bloch's theorem and the magnetic Brillouin zone.

For any lattice vector $\boldsymbol{R}$, the corresponding translation operator is[3]

$$\mathcal{T}(\boldsymbol{R}) := e^{i\boldsymbol{R}\cdot\boldsymbol{p}}, \tag{S36}$$

whose action on a wavefunction is $\mathcal{T}(\boldsymbol{R})\psi(\boldsymbol{r}) = \psi(\boldsymbol{r}+\boldsymbol{R})$. At zero field, $[\mathcal{T}(\boldsymbol{R}), \boldsymbol{p}] = 0$, so lattice translations commute with Hamiltonians involving a periodic potential $H = p^2/(2m) + V(r)$. At constant magnetic field $\boldsymbol{B} = B\hat{z}$, $\mathcal{T}(\boldsymbol{R})$ and $\boldsymbol{\pi} = \boldsymbol{p} + \boldsymbol{A}$ generically fail to commute. To rectify this, we consider the *magnetic translation algebra* [15, 24],

$$\mathcal{M}(\boldsymbol{R}) := e^{-i\xi_{\boldsymbol{R}}(\boldsymbol{r})}\mathcal{T}(\boldsymbol{R}) \quad \text{where} \quad \boldsymbol{A}(\boldsymbol{r}+\boldsymbol{R}) - \boldsymbol{A}(\boldsymbol{r}) = -\boldsymbol{\nabla}\xi_{\boldsymbol{R}}(\boldsymbol{r}). \tag{S37}$$

(Since $\boldsymbol{\nabla} \times [\boldsymbol{A}(\boldsymbol{r}+\boldsymbol{R}) - \boldsymbol{A}(\boldsymbol{r})] = 0$, $\xi_{\boldsymbol{R}}$ is well-defined.) Then

$$[\boldsymbol{\pi}, \mathcal{M}(\boldsymbol{R})]\psi(\boldsymbol{r}) = e^{i\xi_{\boldsymbol{R}}(\boldsymbol{r})}\left[\Big(-i\boldsymbol{\nabla} + i\boldsymbol{\nabla}\Big) + \Big(-\boldsymbol{\nabla}\xi_{\boldsymbol{R}}(\boldsymbol{r}) + \boldsymbol{A}(\boldsymbol{r}) - \boldsymbol{A}(\boldsymbol{r}+\boldsymbol{R})\Big)\right]\psi(\boldsymbol{r}+\boldsymbol{R}) = 0, \tag{S38}$$

i.e. $\xi_{\boldsymbol{R}}$ was chosen so that the magnetic translation operator commutes with the kinetic part of the Hamiltonian.

However, the magnetic translation operators do not always commute among themselves, as

$$\mathcal{M}(\boldsymbol{R}_1)\mathcal{M}(\boldsymbol{R}_2) = \mathcal{M}(\boldsymbol{R}_2)\mathcal{M}(\boldsymbol{R}_1)\zeta, \text{ where } \zeta = e^{i2\pi\Phi/\Phi_0}, \quad \frac{\Phi}{\Phi_0} = \frac{eB|\boldsymbol{R}_1 \times \boldsymbol{R}_2|}{2\pi\hbar}, \tag{S39}$$

To apply Bloch's theorem, the translation operators must mutually commute with themselves, as well as the Hamiltonian. For rational values of the flux

$$\frac{\Phi}{\Phi_0} = \frac{p}{q}, \tag{S40}$$

one has $\zeta^q = 1$. Enlarging the unit cell by a factor of q gives a maximal set of commuting translation operators, such as the one generated by $\mathcal{M}(\boldsymbol{R}_1), \mathcal{M}(\boldsymbol{R}_2)^q$ so that $\zeta = (e^{i2\pi p/q})^q = 1$.[4] The (magnetic) Bloch's theorem is then the statement that $\mathcal{M}(\boldsymbol{R}_1), \mathcal{M}(\boldsymbol{R}_2)$, and H may be simultaneously diagonalized. The eigenvectors take the form $|\psi_{a\boldsymbol{k}}\rangle$ and obey [26]

$$H\,|\psi_{a\boldsymbol{k}}\rangle = \epsilon_{a\boldsymbol{k}}\,|\psi_{a\boldsymbol{k}}\rangle \tag{S41a}$$

$$\mathcal{M}(\boldsymbol{R})\,|\psi_{a\boldsymbol{k}}\rangle = e^{i\boldsymbol{k}\cdot\boldsymbol{R}}\,|\psi_{a,\boldsymbol{k}}\rangle\,. \tag{S41b}$$

where a is a band index, $\boldsymbol{R}$ is an integer combination of of $\boldsymbol{R}_1$ and $q\boldsymbol{R}_2$, and $\boldsymbol{k}$ lives in the *magnetic Brillouin zone*, obtained by folding the normal Brillouin zone over itself q times. The magnetic field gives an extra twist (phase factor) relative to the normal case. By definition, $\mathcal{M}(\boldsymbol{R})\psi_{a\boldsymbol{k}}(\boldsymbol{r}) = e^{-i\xi_{\boldsymbol{R}}(\boldsymbol{r})}\psi_{a\boldsymbol{k}}(\boldsymbol{r}+\boldsymbol{R})$, so

$$\psi_{a\boldsymbol{k}}(\boldsymbol{r}+\boldsymbol{R}) = e^{i\boldsymbol{k}\cdot\boldsymbol{R}+i\xi_{\boldsymbol{R}}(\boldsymbol{r})}\psi_{a\boldsymbol{k}}(\boldsymbol{r}) \tag{S42}$$

$$u_{a\boldsymbol{k}}(\boldsymbol{r}+\boldsymbol{R}) = e^{i\xi_{\boldsymbol{R}}(\boldsymbol{r})}u_{a\boldsymbol{k}}(\boldsymbol{r}) \tag{S43}$$

where the Bloch wavefunction are defined as usual by $u_{a\boldsymbol{k}}(\boldsymbol{r}) := e^{-i\boldsymbol{k}\cdot\boldsymbol{r}}\psi_{a\boldsymbol{k}}(\boldsymbol{r})$. The fact that they satisfy twisted boundary conditions instead of being periodic on the unit cell is a consequence of the magnetic field.

The magnetic Brillouin zone has one other structure. Even though only $\mathcal{M}(q\boldsymbol{R}_2)$ is part of the commuting set, one still has $[H, \mathcal{M}(\boldsymbol{R}_2)] = 0$, so $H(\mathcal{M}(\boldsymbol{R}_2)\,|\psi_{a\boldsymbol{k}}\rangle) = \epsilon_{a\boldsymbol{k}}(\mathcal{M}(\boldsymbol{R}_2)\,|\psi_{a\boldsymbol{k}}\rangle)$. It follows from Eq. (S39) that $\mathcal{M}(\boldsymbol{R}_2)\,|\psi_{a\boldsymbol{k}}\rangle = |\psi_{a(\boldsymbol{k}+\boldsymbol{K}_2/q)}\rangle$, where $\boldsymbol{K}_2$ is the second reciprocal lattice vector [26]. So one need only consider a $1/q$ part of the magnetic Brillouin zone (but there are q times as many bands as the non-magnetic problem at each $\boldsymbol{k}$-point).

C. Matrix Elements

We now specialize to the case of TBG. Following previous work, we choose a rectangular Brillouin zone of size $2Q_x \times Q_y$ with $Q_x = (\sqrt{3}/2)k_\theta$, $Q_y = (3/2)k_\theta$. As Q_xQ_y is half the area of the Moire Brillouin zone, the commensurability condition (S40) becomes

$$\frac{\Phi}{\Phi_0} = \frac{BA_M}{\Phi_0} = \frac{1}{2}\frac{p}{q} \text{ or } Q_xQ_y\ell^2 = 2\pi\frac{q}{p}, \tag{S44}$$

---

[3] We take $e = c = \hbar = 1$ in this section.
[4] Any choice of generators $\mathcal{M}(\boldsymbol{R}_1)^r$, $\mathcal{M}(\boldsymbol{R}_2)^s$ such that $r + s = q$ is permissible.

where $\Phi_0 = h/e = 2\pi\hbar/e$. We adopt the Landau gauge $\boldsymbol{A} = (-By, 0)$ and a magnetic unit cell

$$a_x = \frac{2\pi}{Q_x}; \quad a_y = \frac{2\pi}{Q_y/q} = pQ_x\ell^2 =: p\Delta. \tag{S45}$$

The corresponding magnetic Brillouin zone is

$$\begin{cases} 0 \le k_x \le Q_x \\ 0 \le k_y \le \frac{Q_y}{q}. \end{cases} \tag{S46}$$

Solving Eq. (S37) gives twists $\xi_1(\boldsymbol{r}) = 0$, $\xi_2(\boldsymbol{r}) = a_y x/\ell^2$, so that

$$u(x + a_x, y) = u(x, y) \tag{S47a}$$

$$u(x, y + a_y) = e^{ixa_y/\ell^2} u(x, y). \tag{S47b}$$

We select a basis so that (i) the kinetic part of the Hamiltonian becomes algebraic and (ii) the tunneling terms are k-diagonal in the magnetic Brillouin zone. Let $|\gamma, \sigma, n, Y\rangle$ be the Landau level basis of Eq. (S26), with orbital indices γ and σ running over layer and sublattice respectively. The tunneling terms $T_s e^{-i\boldsymbol{b}_s\cdot\boldsymbol{r}}$ will each induce a change in the guiding center of a multiple of $\Delta = Q_x\ell^2$. We will therefore perform a discrete Fourier transform in steps of Δ. Write

$$Y_{M,j} = k_x\ell^2 + (Mp + j)\Delta, \quad j \in \mathbb{Z}/p\mathbb{Z},\ M \in \mathbb{Z}. \tag{S48}$$

Then define

$$|\gamma, \sigma, n, k_x, k_y, j\rangle = a_x^{-1/2} \sum_{M\in\mathbb{Z}} e^{ik_y(Mp+j)\Delta} |\gamma, \sigma, n, Y = Y_{M,j}\rangle. \tag{S49}$$

The "internal" variable $0 \le k_x < Q_x$ give the x-coordinate of magnetic BZ, and the conjugate variable to M will be $0 \le k_y \le Q_y/q$ (recall $Mp\Delta = Ma_y$ shifts the y coordinate). Finally, j indexes the p-fold covering of the magnetic Brillouin zone.[5] Explicitly, the wavefunctions are

$$\langle \boldsymbol{r}|\gamma, \sigma, n, j, \boldsymbol{k}\rangle = (a_x\ell)^{-1/2} \sum_{M\in\mathbb{Z}} e^{ik_y(Mp+j)\Delta} e^{ixY_{M,j}/\ell^2} \varphi_n([y - Y_{M,j}]/\ell) |\gamma, \sigma\rangle \tag{S50}$$

with normalization $(2\pi)^2\delta(\boldsymbol{k}-\boldsymbol{k}')\delta_{\gamma',\gamma}\delta_{\sigma'\sigma}$. To compute matrix elements, we will make use of the Bloch wavefunctions

$$u_{\gamma,\sigma,n,j,\boldsymbol{k}}(\boldsymbol{r}) = \langle \boldsymbol{r}|\, e^{-i\boldsymbol{k}\cdot\boldsymbol{r}}\, |\gamma, \sigma, n, j, \boldsymbol{k}\rangle = (a_x\ell)^{-1/2} \sum_{M\in\mathbb{Z}} e^{-ik_y[y-(Mp+j)\Delta]} e^{ix(Mp+j)Q_x} \varphi_n([y - Y_{M,j}]/\ell), \tag{S51}$$

which are normalized at each $\boldsymbol{k} = (k_x, k_y)$ as $\int_{\text{UC}} d^2\boldsymbol{r}\ u_{\gamma\sigma nj\boldsymbol{k}}(\boldsymbol{r})^* u_{\gamma'\sigma'mj'\boldsymbol{k}}(\boldsymbol{r}) = \delta_{\gamma\gamma'}\delta_{\sigma\sigma'}\delta_{nm}\delta_{jj'}$, where the integral is over a magnetic unit cell.

We now compute the matrix elements. By design, the kinetic term is simple in terms of Landau levels.

$$h^{\pm} = v_F \boldsymbol{\sigma}_{\pm\theta/2} \cdot \left[(\pi_x, \pi_y) \mp \frac{\hbar}{2}\boldsymbol{q}_0\right] = v_F\sigma^+ e^{\pm i\theta/2}\left[\pi_x - i\pi_y \mp \frac{i\hbar q_0}{2}\right] + \text{h.c.} = \hbar v_F \sigma^+ e^{\pm i\theta/2}\left[\frac{\sqrt{2}}{\ell}\hat{a} \mp \frac{ik_\theta}{2}\right] + \text{h.c.} \tag{S52}$$

The tunneling terms are slightly more involved. The exponential $e^{-i\boldsymbol{b}_s\cdot\boldsymbol{r}}$ is, of course, periodic in Q_x and Q_y. Explicitly, $\boldsymbol{b}_{0,1,2} = (rQ_x, sQ_y)$ for $(r, s) = (0, 0), (1, 1), (-1, 1)$ respectively. Therefore it will be k-diagonal and, as it only involves $\boldsymbol{r}$, the Bloch wavefunctions give the matrix element. We now compute

$$\begin{aligned}
&\langle u_{\gamma'\sigma'mj'\boldsymbol{k}}|\mathcal{T}e^{-i[rQ_x x + sQ_y y]}|u_{\gamma\sigma nj\boldsymbol{k}}\rangle \\
&= (\gamma^+)_{\gamma'\gamma} (\mathcal{T})_{\sigma'\sigma} \sum_{M,M'\in\mathbb{Z}} \int_0^{a_x} \frac{dx}{a_x} \int_0^{a_y} \frac{dy}{\ell} e^{ik_y[y-(M'p+j')\Delta]} e^{-ix[M'p+j']Q_x} e^{-ik_y[y-(Mp+j)\Delta]} e^{ix[Mp+j]Q_x} \\
&\qquad \times \varphi_m([y - Y']/\ell) e^{-i[rQ_x x + sQ_y]} \varphi_n([y - Y]/\ell),
\end{aligned}$$

[5] In fact, one can arrive at this basis purely through symmetry considerations [26].

where $Y' = Y_{M',j'} = k_x\ell^2 + (M'p + j')\Delta$ and similarly for Y. The x-integral gives a δ-function $\delta_{M'M}\delta_{j',j-r}$[6] which leaves

$$\left(\gamma^+\right)_{\gamma'\gamma}(\mathcal{T})_{\sigma'\sigma}\,\delta_{j',j-r}e^{ik_yr\Delta}\sum_M\int_0^{a_y}\frac{dy}{\ell}\varphi_m([y-Y']/\ell)e^{-isQ_yy}\varphi_n([y-Y]/\ell). \tag{S53}$$

We then go to dimensionless variables via the substitution $\tilde{y} = \tilde{y}_{M',j'} = [y - Y']/\ell$ or $y = \ell\tilde{y} + Y'$. Note that $[y - Y]/\ell = \tilde{y} - rQ_x\ell$, so

$$\left(\gamma^+\right)_{\gamma'\gamma}(\mathcal{T})_{\sigma'\sigma}\,\delta_{j',j-r}e^{ik_yr\Delta}\sum_M\int_{\tilde{y}(0)}^{\tilde{y}(a_y)}d\tilde{y}\;\varphi_m(\tilde{y})e^{-isQ_y\ell\tilde{y}}e^{-isQ_yY'}\varphi_n(\tilde{y}-rQ_x\ell). \tag{S54}$$

Using the commensurability relation Eq. (S44), the factor $e^{-isQ_yY'} = e^{-isk_xQ_y\ell^2}e^{-isj'Q_xQ_y\ell^2}$ becomes M-independent. We may then combine the sum and integral to $\sum_M\int_{\tilde{y}(0)}^{\tilde{y}(a_y)}d\tilde{y} = \int_{\mathbb{R}}d\tilde{y}$. Then

$$\left(\gamma^+\right)_{\gamma'\gamma}(\mathcal{T})_{\sigma'\sigma}\,\delta_{j',j-r}e^{ik_yr\Delta}e^{-isk_xQ_y\ell^2}e^{-isj'Q_xQ_y\ell^2}\int_{\mathbb{R}}d\tilde{y}\;\varphi_m(\tilde{y})e^{-isQ_y\ell\tilde{y}}\varphi_n(\tilde{y}-rQ_x\ell). \tag{S55}$$

Turning the coordinate offset into an operator $\varphi_n(\tilde{y} - rQ_x\ell) = e^{-irQ_x\ell\tilde{p}}\varphi_n(\tilde{y})$, the integral is exactly the matrix element $\mathcal{M}_{mn}$ from Eq. (S27) with $\boldsymbol{q} = (rQ_x, sQ_y)$. So

$$\langle u_{\gamma'\sigma'mj'\boldsymbol{k}}|\mathcal{T}e^{-i[rQ_xx+sQ_yy]}|u_{\gamma\sigma nj\boldsymbol{k}}\rangle = \left(\gamma^+\right)_{\gamma'\gamma}(\mathcal{T})_{\sigma'\sigma}\,\delta_{j',(j-r)}e^{ik_yr\Delta}e^{-isk_xQ_y\ell^2}e^{-i[sj-rs/2]2\pi q/p}L_{mn}(z), \tag{S56}$$

again using the commensurability $Q_xQ_y\ell^2 = 2\pi q/p$ and with $L_{mn}(z)$ from Eq. (S34) with $z = \frac{\ell}{\sqrt{2}}(rQ_x + isQ_y)$.

We may now write our model entirely explicitly as

$$H = \int_{MBZ}[d\boldsymbol{k}]\quad h_0(\boldsymbol{k}) + h_1(\boldsymbol{k}) + \sum_{s=0}^{2}(\mathcal{T}_s(\boldsymbol{k}) + h.c.) \tag{S57}$$

where MBZ is the magnetic Brillouin zone, h_0 is the raising and lowering part of the kinetic term and h_1 is the Landau-diagonal part which encodes the offsets of the Dirac points. Using 4×4 matrices for layer-sublattice space, the matrix elements are

$$\langle\gamma',\sigma',m,j',\boldsymbol{k}|h_0(\boldsymbol{k})|\gamma,\sigma,n,j,\boldsymbol{k}\rangle = \epsilon\frac{\sqrt{2}}{\ell k_\theta}\delta_{j'j}\begin{pmatrix} & \eta\sqrt{n}\delta_{m,n-1} & & \\ \eta^*\sqrt{m}\delta_{m,n+1} & & & \\ & & & \eta^*\sqrt{n}\delta_{m,n-1} \\ & & \eta\sqrt{m}\delta_{m,n+1} & \end{pmatrix}_{\gamma'\sigma',\gamma\sigma} \tag{S58a}$$

$$\langle\gamma',\sigma',m,j',\boldsymbol{k}|h_1(\boldsymbol{k})|\gamma,\sigma,n,j,\boldsymbol{k}\rangle = \epsilon\;\delta_{j'j}\delta_{mn}\begin{pmatrix} & i\eta/2 & & \\ -i\eta^*/2 & & & \\ & & & -i\eta^*/2 \\ & & i\eta/2 & \end{pmatrix}_{\gamma'\sigma',\gamma\sigma} \tag{S58b}$$

$$\langle\gamma',\sigma',m,j',\boldsymbol{k}|\mathcal{T}_0(\boldsymbol{k})|\gamma,\sigma,n,j,\boldsymbol{k}\rangle = \delta_{j',j}(\gamma^+)_{\gamma',\gamma}\delta_{mn}(T_0)_{\sigma',\sigma} \tag{S58c}$$

$$\langle\gamma',\sigma',m,j',\boldsymbol{k}|\mathcal{T}_1(\boldsymbol{k})|\gamma,\sigma,n,j,\boldsymbol{k}\rangle = \delta_{j',(j-1)}e^{ik_y\Delta}e^{-ik_xQ_y\ell^2}e^{-i\frac{2\pi q}{p}(j-1/2)}L_{mn}(z_1)(\gamma^+)_{\gamma',\gamma}(T_1)_{\sigma',\sigma} \tag{S58d}$$

$$\langle\gamma',\sigma',m,j',\boldsymbol{k}|\mathcal{T}_2(\boldsymbol{k})|\gamma,\sigma,n,j,\boldsymbol{k}\rangle = \delta_{j',(j+1)}e^{-ik_y\Delta}e^{-ik_xQ_y\ell^2}e^{-i\frac{2\pi q}{p}(j+1/2)}L_{mn}(z_2)(\gamma^+)_{\gamma',\gamma}(T_2)_{\sigma',\sigma}. \tag{S58e}$$

Here $\eta = e^{i\theta/2}$, $z_n = \frac{\ell}{\sqrt{2}}\Big((b_n)_x + i(b_n)_y\Big)$, and $j, j' \in \mathbb{Z}/p\mathbb{Z}$, and the dimensionful factor for the kinetic term is $\epsilon := \hbar v_F k_\theta = \frac{3}{2}t_{NN}k_\theta$, where $t_{NN} \approx 2.83\,\text{eV}$ is the nearest neighbor hopping of graphene. To add an hBN potential to the bottom layer, one can add a term $(-\Delta_{\text{hBN}}/2)\delta_{mn}\delta_{j'j}(\gamma^z + \gamma^0)\sigma^z$. It is convenient to express factors in terms of the dimensionless ratio p/q whenever possible. To this end, the commensurability relation (S44) implies the identities

$$\frac{3\sqrt{3}}{4}k_\theta^2\ell^2 = \frac{2\pi q}{p},\qquad \frac{\ell Q_x}{\sqrt{2}} = \left(\frac{\pi}{\sqrt{3}}\frac{q}{p}\right)^{\frac{1}{2}},\qquad \frac{\ell Q_y}{\sqrt{2}} = \left(\sqrt{3}\pi\frac{q}{p}\right)^{\frac{1}{2}}. \tag{S59}$$

[6] Although M can 'roll over' for some values of j, j', the answer is identical.

Thus we have arrived at a completely explicit expression for the Bistritzer-MacDonald model in a finite field.

Let us give a few notes on numerical implementation. Technically, the Hamiltonian is a (countably) infinite dimensional operator at each $\boldsymbol{k}$ point. However, as we are only interested in low-energy properties of the model (i.e. the narrow bands), we may take a Landau level cutoff of $n, m < N$, where N is a few hundred (scaling with q). This gives a spurious zero mode at LL N, which may be gapped out by adding an appropriate potential to the largest Landau level [20]. This gives $H(\boldsymbol{k})$ of dimension $4pN$. The Laguerre factor $L_{mn}(z)$ decays (exponentially) quickly with $|m-n|$, so only a small number of matrix elements are above numerical precision (the factorials factors must be treated carefully to avoid floating point overflows and underflows).

D. Non-Abelian Quantum Geometric Tensor

As the basis of Landau levels has intrinsic curvature, the standard formulas for computing the Berry curvature and quantum geometric tensor must be modified. We seek a (numerically stable) formula for the Berry curvature and quantum geometric tensor. In the simple case of a single band, [22] showed that the $U(1)$ berry curvature has a correction $\mathcal{F} = \mathcal{F}_{\text{numerical}} - 1/p$. We must consider the $U(N)$ case, whereupon the answer is no longer a sum of the numerical answer plus an analytical correction.

The $U(N)$ Berry curvature may be computed as the holonomy of an Wilson loop around an infinitesimal square [27]

$$e^{i\mathcal{F}(\boldsymbol{k})\Delta k_x \Delta k_y} = W_{\square}(k) = U_{\boldsymbol{k},\boldsymbol{k}+\boldsymbol{\Delta}_x} U_{\boldsymbol{k}+\boldsymbol{\Delta}_x,\boldsymbol{k}+\boldsymbol{\Delta}_x+\boldsymbol{\Delta}_y} U_{\boldsymbol{k}+\boldsymbol{\Delta}_x+\boldsymbol{\Delta}_y,\boldsymbol{k}+\boldsymbol{\Delta}_y} U_{\boldsymbol{k}+\boldsymbol{\Delta}_y,\boldsymbol{k}} \tag{S60}$$

where $\boldsymbol{\Delta}_\alpha = \Delta k_\alpha \boldsymbol{e}_\alpha$ and the *link variables* are defined as

$$[U_{\boldsymbol{k},\boldsymbol{k}'}]_{ab} := \langle U_a(\boldsymbol{k}) | U_b(\boldsymbol{k}') \rangle \,, \tag{S61}$$

in terms of the $U_a(\boldsymbol{k})$, the (normalized) Bloch wavefunction for band a. Due to the non-Abelian connection, the matrix elements of $W_\square$ are gauge covariant, but its eigenvalues — and hence its trace and determinant — are gauge-invariant. In practice, $U_a(\boldsymbol{k})$ is evaluated only on a discrete grid within the Brillouin zone, and Eq. (S60) provides a numerically stable formula for the Chern number and other geometrical quantities such as $\sigma[\mathcal{F}]$.

The wrinkle from the magnetic field is that the link variables have both an analytic part from the Landau levels as well as a numerical part. Let the eigenstates be given by

$$|U_a(\boldsymbol{k})\rangle = \sum_I \psi_a^I \, |u_I(\boldsymbol{k})\rangle \,, \tag{S62}$$

where $I = (\gamma, \sigma, n, j)$ is a multi-index, ψ_a^I are the numerical eigenstates, and $|u_I(\boldsymbol{k})\rangle$ are the Bloch basis vectors defined in Eq. (S51). Consider the form of the k-derivative:

$$\partial_{k_\alpha} |u^a(\boldsymbol{k})\rangle = \sum_I \left(\partial_{k_\alpha} \psi_a^I\right) |u_I\rangle + \psi_a^I \, |\partial_{k_\alpha} u_I\rangle \,. \tag{S63}$$

In most tight-binding models, the second term vanishes, but the magnetic field ensures it does not here. The link variable is then a quadratic form

$$[U_{\boldsymbol{k},\boldsymbol{k}'}]_{ab} = \sum_{IJ} \langle u_I(\boldsymbol{k}) | u_J(\boldsymbol{k}') \rangle \, \overline{\psi}_a^I \psi_b^J =: \sum_{I,J} \overline{\psi}_a^I \mathcal{T}(\boldsymbol{k},\boldsymbol{k}')_{IJ} \, \psi_b^J \tag{S64}$$

where we have used $\overline{\psi}$ to denote the complex conjugate. We may compute $\mathcal{T}$ analytically,

$$\begin{aligned}
\mathcal{T}(\boldsymbol{k},\boldsymbol{k}+\boldsymbol{q}) &= \langle u_{\gamma,\sigma,n,j,\boldsymbol{k}} | u_{\gamma',\sigma',m,j',\boldsymbol{k}+\boldsymbol{q}} \rangle \\
&= \delta_{\gamma\gamma'}\delta_{\sigma\sigma'} \int_0^{a_x} \frac{dx}{a_x} \int_0^{a_y} dy \sum_{M,M'\in\mathbb{Z}} e^{ik_y[y-(Mp+j)\Delta]} e^{-ix(Mp+j)Q_x} \ell^{-1/2} \varphi_n([y-Y]/\ell) \\
&\qquad \times \; e^{-i(k_y+q_y)[y-(M'p+j')\Delta]} e^{ix(M'p+j')Q_x} \ell^{-1/2} \varphi_m([y-Y'-\ell^2 q_x]/\ell) \\
&= \delta_{\gamma\gamma'}\delta_{\sigma\sigma'}\delta_{jj'} \sum_{M\in\mathbb{Z}} \int_0^{a_y} \frac{dy}{\ell} e^{-iq_y[y-(M'p+j')\Delta]} \varphi_n([y-Y]/\ell) \varphi_m([y-Y-\ell^2 q_x]/\ell) \\
&= \delta_{\gamma\gamma'}\delta_{\sigma\sigma'}\delta_{jj'} \int_{\mathbb{R}} d\tilde{y} \; \varphi_n(\tilde{y}) e^{-iq_y[\ell\tilde{y}+\ell^2 k_x]} \varphi_m(\tilde{y} - q_x\ell) \\
&= \delta_{\gamma\gamma'}\delta_{\sigma\sigma'}\delta_{jj'} e^{-i\ell^2 q_y k_x} \int_{\mathbb{R}} d\tilde{y} \; \varphi_n(\tilde{y}) e^{-iq_y\ell\tilde{y}} e^{-iq_x\ell\tilde{p}_y} \varphi_m(\tilde{y})
\end{aligned}$$

Again the matrix element involves $\mathcal{M}_{nm}(\boldsymbol{q})$, so

$$\mathcal{T}(\boldsymbol{k}, \boldsymbol{k}+\boldsymbol{q}) = \delta_{\gamma\gamma'}\delta_{\sigma\sigma'}\delta_{jj'}e^{-ik_xq_y\ell^2}e^{-iq_xq_y\ell^2/2}L_{nm}(z); \quad z := \frac{\ell}{\sqrt{2}}(q_x + iq_y). \tag{S65}$$

This is almost independent of $\boldsymbol{k}$. Consider a grid of $N_x \times N_y$ points with separation $q_x = Q_x/N_x$ and $q_y = (Q_y/q)/N_y$. Then only two matrices are needed:

$$\mathcal{T}_x := \mathcal{T}(\boldsymbol{k}, \boldsymbol{k}+q_x\boldsymbol{e}_x) = \delta_{\gamma\gamma'}\delta_{\sigma\sigma'}\delta_{jj'}L_{nm}(z_1); \qquad z_1 = \frac{\ell Q_x}{\sqrt{2}N_x} = \left(\frac{\pi q}{\sqrt{3}p}\right)^{1/2}\frac{1}{N_x}, \tag{S66}$$

$$\mathcal{T}_y := \mathcal{T}(\boldsymbol{k}, \boldsymbol{k}+q_y\boldsymbol{e}_y)e^{i\ell^2q_yk_x} = \delta_{\gamma\gamma'}\delta_{\sigma\sigma'}\delta_{jj'}L_{nm}(z_2); \qquad z_2 = i\frac{\ell Q_y/q}{\sqrt{2}N_y} = i\left(\frac{\sqrt{3}\pi}{qp}\right)^{1/2}\frac{1}{N_y}. \tag{S67}$$

Th "extra" exponential factor in $\mathcal{T}_y$ is in fact the band-diagonal correction found in [22]. To see this, note

$$e^{-i\ell^2q_y(k_x+q_x)}e^{-i\ell^2(-q_y)k_x} = e^{-i\ell^2q_xq_y} = e^{-i\frac{2\pi}{N_xN_yp}}. \tag{S68}$$

As $\operatorname{tr}\mathcal{F} = \operatorname{Im}\log\det W_\square$, integrating this factor gives a correction to $(2\pi)^{-1}\int\operatorname{tr}\mathcal{F}$ by $1/p$ times the number of bands. In full, we may compute

$$W_\square = e^{-i\frac{2\pi}{N_xN_yp}}(V_x)_{\boldsymbol{k},\boldsymbol{k}+\boldsymbol{\Delta}_x}(V_y)_{\boldsymbol{k}+\boldsymbol{\Delta}_x,\boldsymbol{k}+\boldsymbol{\Delta}_x+\boldsymbol{\Delta}_y}(V_x^\dagger)_{\boldsymbol{k}+\boldsymbol{\Delta}_x+\boldsymbol{\Delta}_y,\boldsymbol{k}+\boldsymbol{\Delta}_y}(V_y^\dagger)_{\boldsymbol{k}+\boldsymbol{\Delta}_y,\boldsymbol{k}} \tag{S69}$$

with $(V_\alpha)_{\boldsymbol{k},\boldsymbol{k'}} = \sum_{IJ}\overline{\psi}^I_a(\boldsymbol{k})\mathcal{T}_\alpha\psi^J_b(\boldsymbol{k'})$ and $(V_\alpha^\dagger)_{\boldsymbol{k},\boldsymbol{k'}}$ is the same with $\mathcal{T}_\alpha^\dagger$. Eq. (S69) provides an efficient and numerically stable formula for the Berry curvature. For $\sigma[\mathcal{F}]$, one uses $\operatorname{tr}[\mathcal{F}^2] = (2\pi)^{-2}\sum_a(\log\sigma_a)^2$, where σ_a are the eigenvalues of $W_\square$ (which are gauge-invariant). The quantum metric may be computed similarly with the Wilson loop $\boldsymbol{k} \to \boldsymbol{k} + q_\alpha\boldsymbol{e}_\alpha \to \boldsymbol{k}$.

---

[1] R. Roy, Physical Review B **90**, 165139 (2014).
[2] S. Parameswaran, R. Roy, and S. L. Sondhi, Physical Review B **85**, 241308 (2012).
[3] X.-L. Qi, Physical review letters **107**, 126803 (2011).
[4] T. S. Jackson, G. Möller, and R. Roy, Nature communications **6**, 1 (2015).
[5] M. Goerbig, The European Physical Journal B **85**, 1 (2012).
[6] M. Claassen, C. H. Lee, R. Thomale, X.-L. Qi, and T. P. Devereaux, Physical review letters **114**, 236802 (2015).
[7] P. J. Ledwith, G. Tarnopolsky, E. Khalaf, and A. Vishwanath, Physical Review Research **2**, 023237 (2020).
[8] C. Repellin and T. Senthil, Physical Review Research **2**, 023238 (2020).
[9] A. Abouelkomsan, Z. Liu, and E. J. Bergholtz, Physical review letters **124**, 106803 (2020).
[10] P. Wilhelm, T. C. Lang, and A. M. Läuchli, Physical Review B **103**, 125406 (2021).
[11] T. Scaffidi and G. Möller, Physical review letters **109**, 246805 (2012).
[12] A. Bohm, A. Mostafazadeh, H. Koizumi, Q. Niu, and J. Zwanziger, *The Geometric phase in quantum systems: foundations, mathematical concepts, and applications in molecular and condensed matter physics* (Springer Science & Business Media, 2013).
[13] B. Mera and T. Ozawa, arXiv preprint arXiv:2103.11583 (2021).
[14] M. M. Fogler, arXiv preprint arXiv:cond-mat/0111001 (2001).
[15] T. Ozawa and B. Mera, arXiv preprint arXiv:2103.11582 (2021).
[16] S. Girvin, A. MacDonald, and P. Platzman, Physical review letters **54**, 581 (1985).
[17] R. Bistritzer and A. H. MacDonald, Proceedings of the National Academy of Sciences **108**, 12233 (2011).
[18] S. Carr, S. Fang, Z. Zhu, and E. Kaxiras, Phys. Rev. Research **1**, 013001 (2019).
[19] D. R. Hofstadter, Physical review B **14**, 2239 (1976).
[20] R. Bistritzer and A. MacDonald, Physical Review B **84**, 035440 (2011).
[21] Y.-H. Zhang, H. C. Po, and T. Senthil, Physical Review B **100**, 125104 (2019).
[22] K. Hejazi, C. Liu, and L. Balents, **100**, 035115.
[23] A. H. Macdonald, arXiv preprint cond-mat/9410047 (1994).
[24] J. Zak, Physical Review **134**, A1602 (1964).
[25] J. Zak, Physical Review **134**, A1607 (1964).
[26] J. Zak, Physical Review **136**, A776 (1964).
[27] T. Fukui, Y. Hatsugai, and H. Suzuki, Journal of the Physical Society of Japan **74**, 1674 (2005).
